# Bilaterale Irisimplantatexplantation bei Pigmentdispersionssyndrom (PDS) und Hornhautdekompensation nach kosmetischer Irisimplantation (BrightOcular)

**DOI:** 10.1007/s00347-020-01040-6

**Published:** 2020-02-05

**Authors:** Kleopatra Varna-Tigka, Franziska Löffler, Thomas Kohnen

**Affiliations:** Klinik für Augenheilkunde, Goethe-Universität Frankfurt, Theodor-Stern-Kai 7, 60590 Frankfurt am Main, Deutschland

**Keywords:** Kosmetische Irisimplantate, Pigmentdispersionsglaukom, Hornhautödem, Glaukom, Irisfarbenänderung, Cometic iris implants, Pigment dispersion glaucoma, Corneal edema, Glaukoma, Iris color change

## Abstract

Wir berichten über eine 27-jährige Patientin mit Pigmentdispersionssyndrom und Hornhautdekompensation in beiden Augen nach Implantation eines Irisimplantates (BrightOcular, Anaheim, CA, USA) zur Änderung der Augenfarbe. Drei Jahre nach Implantation der künstlichen Iris an einer Augenklinik in Indien erfolgte die konsekutive Entfernung der Implantate bei erhöhtem Risiko der Entwicklung eines Pigmentdispersionsglaukoms und Gefahr der Hornhautdekompensation. Postoperativ zeigten sich traumatische, mittelweite und irreguläre Pupillen.

## Abstract (en)

This article reports the case of a 27-year-old female patient with pigment dispersion syndrome (PDS) and corneal edema in both eyes after cosmetic iris implantation (BrightOcular, Anaheim, CA, USA) in an Indian eye clinic in order to change the eye color. Explantation of the implants was recommended 3 years after implantation and after developing PDS due to the high risk of developing glaucoma and because of corneal decompensation due to loss of corneal endothelial tissue. Postoperatively, traumatic, middle wide and irregular pupils were present.

## Anamnese

Wir berichten über eine 27-jährige Patientin türkischer Abstammung, die sich mit Lichtempfindlichkeit, Fremdkörper- und Trockenheitsgefühl sowie Rötung an beiden Augen erstmalig im Mai 2018 in unserer Klinik vorstellte. Drei Jahre zuvor habe sie sich aus kosmetischen Gründen für das Einsetzen von Irisimplantaten (BrightOcular, Anaheim, CA, USA) entschieden, um die Augenfarbe zu ändern (von braun zu blau). Die Implantation sei an einer Augenklinik in Indien erfolgt.

**Figure 1:**
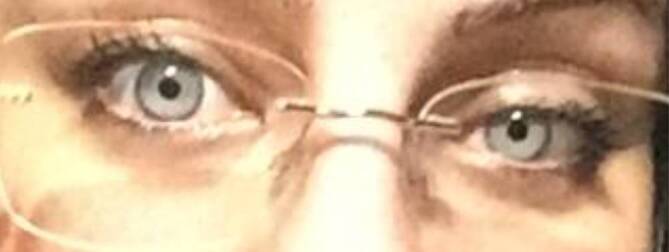

Abgesehen von diesem Eingriff sowie einer mittelgradigen Myopie war die ophthalmologische Anamnese unauffällig. Zur Korrektur der Fehlsichtigkeit trug die Patientin eine Fernbrille sowie Kontaktlinsen (Abb. 1).

## Befund

**Figure 2:**
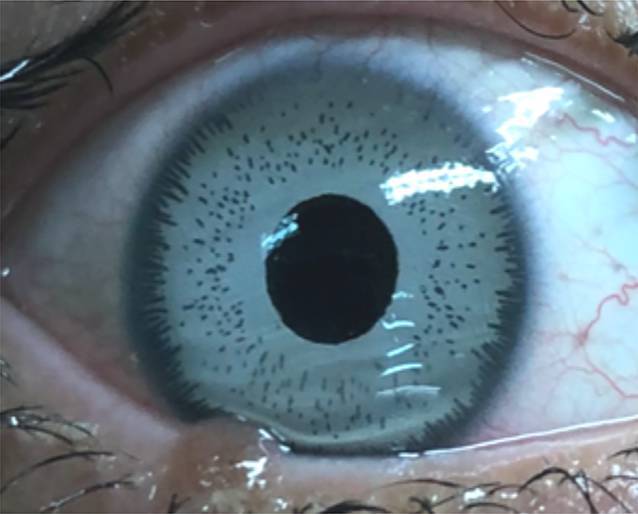

Der Visus betrug an beiden Augen korrigiert 0,8. Es fanden sich ein leichtes Hornhautödem mit flacher Vorderkammer sowie Pigmentbeschläge an der vorderen Linsenkapsel sowie am Endothel. Die hellblauen Irisimplantate waren zentriert. Der Pupillarsaum war durch die zentrale Öffnung im Implantat nur teilweise sichtbar; die Pupillen spielten (Abb. 2). Es fand sich links eine dezente Linsentrübung. Der intraokulare Druck war an beiden Augen im oberen Normbereich. Lider und Bindehaut stellten sich unauffällig dar.

Der Fundusbefund in Miosis war ebenfalls unauffällig mit beidseitiger physiologischer Papillenexkavation und guten Fundusreflexen. Es fand sich eine Endothelzelldichte von 2816 und 1301 Zellen/mm^2^ am rechten bzw. linken Auge. Die Hornhautdicke betrug rechts 542 µm und links 558 µm.

## Therapie und Verlauf

Aufgrund des hohen Risikos eines Pigmentdispersionsglaukoms und weiterer Hornhautdekompensation mit Gefahr der Notwendigkeit einer Hornhauttransplantation wurde die sofortige Explantation der Implantate empfohlen. Insbesondere am linken Auge betonten wir zudem das Risiko einer Kataraktentwicklung. Die Patientin wünschte zunächst Bedenkzeit und stellte sich einige Monate später zur Planung der beidseitigen Irisimplantatexplantation erneut vor. Die ambulante Operation (linkes Auge zuerst) erfolgte an 2 konsekutiven Terminen im Abstand von 1 Woche.

## Operation

Die Patientin erhielt präoperativ 250 mg Acetazolamid (Glaupax®) oral sowie Pilocarpin 1 %ige Augentropfen. Die lokale Anästhesie mit Proparakain-POS 0,5 % ergänzten wir mit einer Peribulbäranästhesie (5 ml Mischung aus Bupivacain und Lidocain).

**Figure 3:**
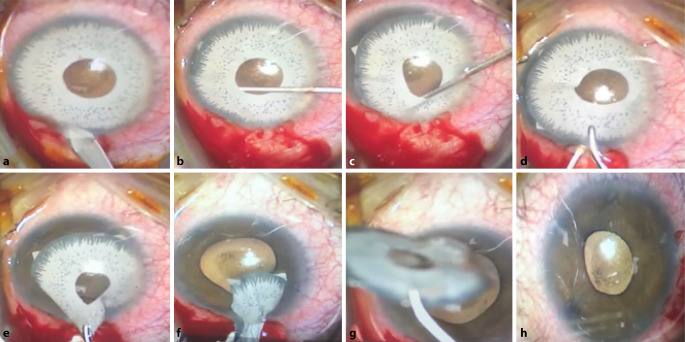

Nach Desinfektion und steriler Abdeckung wurden von einem erfahrenen Operateur (TK) eine temporale korneale Tunnelinzision sowie 2 Parazentesen angelegt. Die Vorderkammer wurde mit einer viskoelastischen Substanz vertieft. Mit Spatel und Pushpull wurden die insgesamt 5 Abstandshalter, die sich im Kammerwinkel abstützen, aus diesem befreit und die Synechien zwischen Implantat und Iris gelöst. Das Implantat konnte gerollt im Ganzen über den Hauptschnitt entfernt werden. Anschließend wurde Acetylcholin zur Verengung der Pupillen in die Vorderkammer eingegeben und die viskoelastische Substanz abgesaugt, die Inzisionen wurden mit Hydratation verschlossen (Abb. 3). Das Auge wurde steril abgedeckt und eine Therapie mit 250 mg Acetazolamid (Glaupax®) 2‑mal täglich eingeleitet. Lokal erhielt die Patientin Dorzolamid 20 mg/Timolol 5 mg Augentropfen (Cosopt®) 2‑mal täglich sowie 1 mg Dexamethason/3,5 mg Neomycin/6000 I.E. Polymyxin‑B (ISOPTO-MAX®) und Nepafenac (Nevanac®) 1 mg/ml Augentropfen jeweils 3‑mal täglich.

## Verlauf

**Figure 4:**
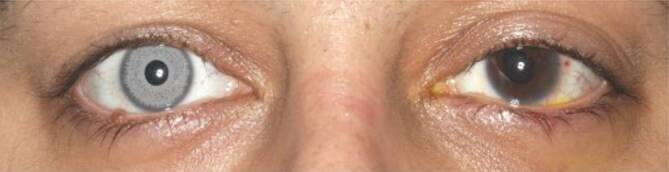

**Figure 5:**
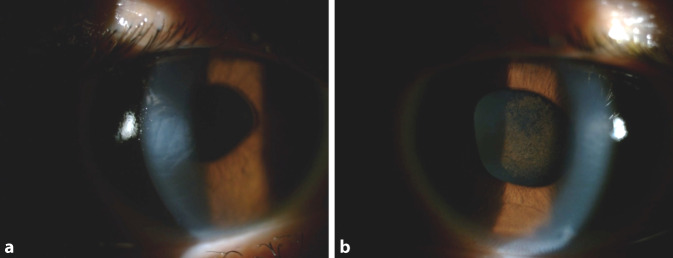

**Figure 6:**
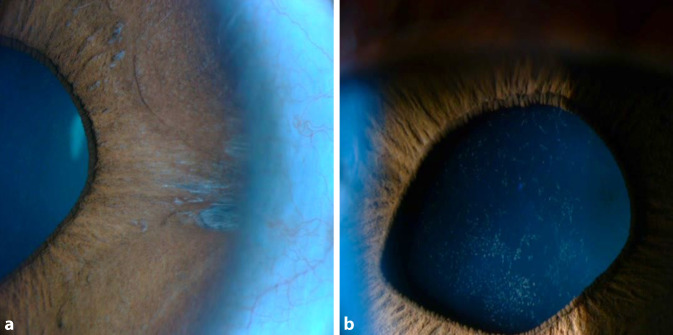

Am jeweils ersten postoperativen Tag betrug der korrigierte Visus an beiden Augen 0,63 (Abb. 4). Unter der lokalen und systemischen Therapie lag der Druck bei 19 mm Hg bzw. 24 mm Hg am rechten bzw. linken Auge. Beidseitig fand sich ein stromales Hornhautödem mit Descemet-Falten sowie Pigmentbeschlägen am Endothel (Abb. 5a). Pigmentablagerungen fanden sich auch zentral auf der Vorderkapsel der Linse durch den Pigmentabtrieb der Irisprothese (Abb. 5b). Fünf periphere Irisdefekte blieben dort zurück, wo die Abstandshalter des Implantats befestigt waren (Abb. 6a). Die Pupillen waren entrundet (Abb. 6b). Eine Woche nach Explantation zeigte die Endothelzellzahlmessung rechts 2633 Zellen/mm^2^, links 799 Zellen/mm^2^.

Die antiglaukomatöse Therapie wurde zunächst belassen, im zeitnahen Verlauf jedoch reduziert. Ein Monat postoperativ zeigte sich eine Stabilisierung des Druckes an beiden Augen bei 24 mm Hg ohne lokale oder systematische Therapie. Drei Monate nach Explantation hatte die Patientin trotz deutlich reduzierter Lichtempfindlichkeit subjektiv keine Beschwerden mehr. Die Befunde zeigten einen stabilen korrigierten Visus beider Augen von 0,8. Der intraokuläre Druck lag im oberen Normbereich bei 21 mm Hg am rechten und 19 mm Hg am linken Auge. Die Endothelzellzahlmessung ergab rechts und links 2267 bzw. 1225 Zellen/mm^2^. Der photopische Pupillendurchmesser war am rechten Auge 4,7 mm und am linken Auge 4,6 mm.

## Diskussion

Es gibt diverse intraokulare Implantate, die sich zur Behandlung von Irisdefekten oder Iriserkrankungen wie Aniridie, Iriskolobomen, okulärem Albinismus oder Photophobie eignen [1, 2]. Die medizinische Indikation solcher Implantate ist die Rekonstruktion des anatomischen Defekts oder die Erhöhung der Lebensqualität im Sinne von Elimination der Lichtempfindlichkeit und Verbesserung der Qualität des Sehens [3]. Implantate wie die Morcher® GmbH (Stuttgart, Deutschland), die HumanOptics (Artificialiris, Erlangen, Deutschland) oder die Ophtec BV-Linsen (Groningen, Netherlands) sind in Europa und/oder in den USA zugelassen. Im Jahr 2006 kam erstmals die Idee der permanenten Irisfarbenänderung aus kosmetischen Gründen [4–16] auf. Die ersten allein kosmetischen Irisimplantate wurden von Kahn Medical Devices Corp. in Panama City US 2006 erfunden und patentiert (Patent 2006#7025781 2B). Die Implantate hatten einen Durchmesser von 15 mm mit einem zentralen Loch in der Pupillarebene von ca. 3,5 mm Durchmesser und einer Dicke von ca. 0,16 mm. Sie waren aus Silikon, und es gab sie in 3 verschiedenen Farben: haselnussfarben, blau und grün. Die Implantate hatten periphere Löcher – sprich Iridektomien – verschiedener Größen, und die Oberfläche bzw. die Rückfläche nach Analysen mit dem Elektromikroskop war sehr irregulär. Laut Operateur und Erfinder wurden bis 2010 mehr als 700 Operationen in Panama durchgeführt. Seither sind diese Implantate nicht mehr auf dem Markt verfügbar.

**Figure 7:**
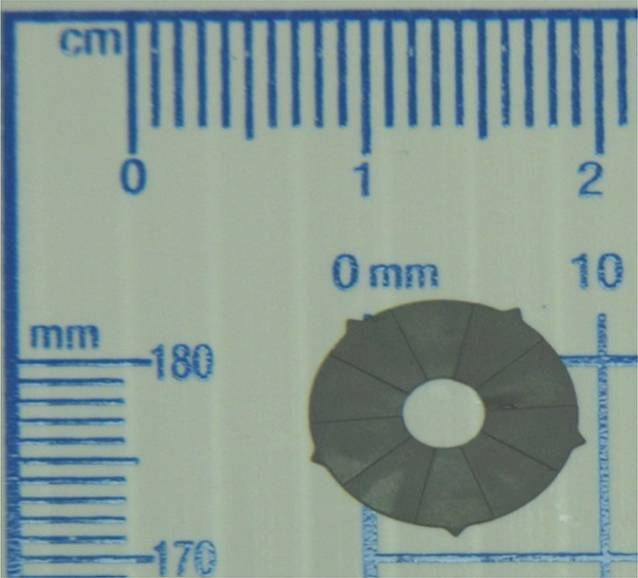

Das BrightOcular (Anaheim, CA, USA) gehört zur zweiten Generation solcher Implantate. Sie wurden von der Firma Stellar Devices in New York, NY, USA, 2012 erfunden und patentiert (Patent 2012#8197540) [17, 18]. Das Implantat hat einen Durchmesser von 11,5–13,5 mm mit einem zentralen Loch von ca. 3,5 mm sowie eine Dicke von 0,3–0,5 mm. In der Peripherie hat es 5 abstehenden Ecken für das Abstützen im Kammerwinkel mit einer Dicke von 0,12–0,14 mm jeweils und einer Länge von 0,8 mm. Die posteriore Fläche des Implantates ist so geformt, dass 10 radiäre Strukturen für einen besseren Abfluss des Kammerwassers sorgen (Abb. 7). Das Implantat besteht aus einem für das Auge nicht toxischen Material. Die Implantation von BrightOcular (Anaheim, CA, USA) wird aktuell in folgenden Ländern durchgeführt: Libanon, Tunesien, Jordanien, Türkei, Indien, China, Syrien, Mexiko, Costa Rica, Albanien. In den USA, Europa und Großbritannien hat dieses Implantat keine Zulassung.

Kosmetische Implantate sind mit einem erhöhten Komplikationsrisiko verbunden [4–16]. Die Befunde reichen von einem erhöhten Endothelzellverlust bis hin zur Hornhautdekompensation, Irisatrophie, Pupillenirregularitäten mit Störungen der Pupillomotorik, frühzeitiger Kataraktentwicklung, anterioren Synechien, sekundärem Winkelblock mit Tensioentgleisung und Pigmentdispersionsglaukom, Uveitis-Glaukom-Hyphäma-Syndrom und glaukombedingter Neuritis nervi optici. Auch andere Komplikationen wie die Entstehung eines zystoiden Makulaödems, zentraler Venenverschluss und traumatische Schädigung des Endothels sowie des Trabekelmaschenwerkes sind möglich. Aufgrund der erhöhten Morbidität ist ein solcher kosmetischer Eingriff insgesamt nicht ratsam. In der Literatur finden sich zahlreiche Fallberichte zu den Risiken von rein kosmetisch verwendeten Irisprothesen, meist ohne positives Outcome [4–16]. Komplikationen können zu verschiedenen Zeitpunkten auftreten, beispielsweise während der In- und Explantation, während der Tragezeit, jedoch unter Umständen auch erst nach Explantation. Die Explantation des Implantats ist bei Auftreten von Komplikationen die einzige sinnvolle Therapiemöglichkeit. Die Wahrscheinlichkeit für Folgeoperationen bei Komplikationen ist zudem hoch. In der Literatur wurde die früheste Explantation schon 3 Wochen nach der initialen Implantation angegeben; die späteste Explantation nach 5 Jahren [19]. In unserem Fall, 3 Jahre nach der initialen Operation, wurde das Transplantat als Ganzes („one-piece“) über einen 2,2 mm temporalen Hauptschnitt entfernt. Ebenso ist die intraokulare Teilung des Transplantates („slicing-the-pie“) möglich. Andere Chirurgen wählten einen größeren Hornhautschnitt (5–6 mm), um das Transplantat in einem Stück zu entfernen [20, 21]. Alle Verfahren der Explantation sollten minimalinvasiv sein, um das Hornhautendothel zu schonen. Anschließend sollten engmaschige Verlaufskontrollen erfolgen, v. a. wenn ein signifikanter Endothelzellverlust oder eine glaukomatöse Erkrankung vorliegt.

Unsere Patientin hatte trotz milder Symptomatik einen signifikanten Endothelzellverlust links und eine ausgeprägte Pigmentdispersion. Die Ergebnisse der Endothelzellzahlmessung schwankten aufgrund des leichten Hornhautödems. Glücklicherweise normalisierte sich der Augeninnendruck, und es fand sich keine glaukomatöse Schädigung. Trotz derzeit stabiler Verhältnisse können wir zukünftige Probleme oder Folgeeingriffe nicht ausschließen.

## Fazit für die Praxis

Die permanente kosmetische Irisfarbänderung mit Implantaten ohne medizinische Indikation ist mit erhöhtem Komplikationsrisiko für das Auge assoziiert. Von einer solchen Operation muss daher abgeraten werden. Insbesondere im Hinblick auf ein Pigmentdispersionssyndrom, das schwer zu behandelnde Pigmentdispersionsglaukom und zunehmenden Endothelzellschaden sollten Vorderkammer- und Kammerwinkel-gestützte Implantate so früh wie möglich entfernt und die Patienten auch nach der Explantation engmaschig kontrolliert werden.
